# Collagen-depletion strategies in dentin as alternatives to the hybrid layer concept and their effect on bond strength: a systematic review

**DOI:** 10.1038/s41598-022-17371-0

**Published:** 2022-07-29

**Authors:** António H. S. Delgado, Madalena Belmar Da Costa, Mário Cruz Polido, Ana Mano Azul, Salvatore Sauro

**Affiliations:** 1 Centro de Investigação Interdisciplinar Egas Moniz (CiiEM), Instituto Universitário Egas Moniz (IUEM), Campus Universitário, Quinta da Granja, Monte de Caparica, 2829-511 Almada, Portugal; 2 Division of Biomaterials and Tissue Engineering, UCL Eastman Dental Institute, University College London, London, UK; 3 Dental Biomaterials and Minimally Invasive Dentistry, Department of Dentistry, Cardenal Herrera-CEU University, CEU Universities, Valencia, Spain

**Keywords:** Biomedical materials, Oral diseases

## Abstract

Strategies aiming to improve the longevity of resin–dentin adhesive interface developed so far have only been able to retard the problem. Different approaches are thus needed. The objective of this review was to determine whether the use of collagen-depletion strategies after acid-etching procedures may improve the bond strength of resin-based materials to dentin. A systematic review was planned following 2021 PRISMA statement guidelines, with a search strategy performed in five electronic databases: PubMed/Medline, Scopus, EMBASE, SciELO and IADR Abstract Archive (last search: 17/01/2022). Inclusion criteria encompassed studies which evaluated a collagen-depletion strategy in acid-etched human dentin and tensile/shear bond strength tests. Risk of bias assessment was carried out by two reviewers, working independently on an adapted five-domain risk of bias (RoB) checklist for laboratory studies. Results were synthesized qualitatively, as a meta-analysis was not possible due to limited number of studies and their RoB. A total of eight studies were eligible for inclusion in the systematic review after inclusion/exclusion criteria application. Out of these, two evaluated the effect of using NaOCl followed by an antioxidant, and the remaining six evaluated different enzymatic treatments (bromelain, chondroitinase ABC, papain, and trypsin). None of the studies reported a decrease of bond strength when a collagen-depletion strategy was used, in comparison to traditional hybrid layers (control). All enzymatic treatment studies which respected the inclusion criteria improved the bond strength to dentin. Some specific collagen-depletion strategies seem to play a favorable role in improving immediate bond strengths to dentin. Further research with sound methodology is required to consolidate these findings, since limitations in RoB and a low number of studies were found. The assessment of further proteolytic agents and long-term outcomes is also required.

## Introduction

Compositional differences between enamel and dentin, such as the absence of a highly sensitive and degradable organic matrix, are responsible for distinct predictability in restorative success^1,2^. In the case of dentin, adhesive procedures rely on the formation of a hybrid layer (HL), a dogma in adhesive dentistry first coined by Nakabayashi in 1982, known today to most important mechanism to secure micromechanical retention in such a substrate^2–4^. While in enamel a simplified and resistant bonding mechanism is tangible, considering that resin monomers are easily drawn into deep mineralized pits and pores, formed through acid etching^5,6^, in dentin, the HL formed within an intricate and complex collagen has an inevitable expiry-date stamp. The inconvenient truth is that virtually all HLs are bound to fail with time.

Indeed, it has been already argued that the foundation stone on which adhesion to dentin was set, is flawed^7^. On the nanoscale, complete envelopment of collagen fibrils by monomers, closing and filling in all spaces, is practically unattainable^8^. Furthermore, to even have a chance in penetrating the interfibrillar spaces of the collagen network, methacrylic acid-based monomers of relative hydrophilicity and low-viscosity must be used; this condemns them to future hydrolysis^9,10^. Thus, to summarize, the existence of exposed collagen, unfilled spaces and water inherently present within dentinal collagen, all either or simultaneously lead to hydrolysis and endogenous enzymatic activity, ultimately responsible for interface degradation^2,7,11^. These reasons led Bertassoni et al. to point out that the resin-dentin interface, from a molecular perspective, is the antithesis of successful bonding^7^.

While most recent research focuses on dealing with this problem by modifying the collagen^12–14^, developing anti-enzymatic strategies, to be used as pre-treatments or as functional components included in dental adhesives^15–17^, or even formulate degradation-resistant polymers^18,19^, it is key to understand that these strategies offer a one-sided solution to a multifactorial problem. While these strategies may improve the outcomes short-term, long-term, either the enzymatic or hydrolytic degradation pathway will inevitably occur, if the organic content remains^5^. Hence, a different approach may be needed.

Several researchers in the late 1990s and early 2000s contested the need for having collagen within the HL at all^20,21^. This would possibly solve the problems mentioned above. In fact, a considerable number of studies found that collagen-depletion by virtue of deproteinizing agents such as sodium hypochlorite (NaOCl) could increase the bond strength or improve interfacial properties of adhesives to dentin^20–23^. NaOCl, as reported by these authors, was able to dissolve most of the organic portion, leaving a mineral-rich layer easily infiltrated by resin monomers. However, a number of studies also found that these results were adhesive-dependent, while arguing that the collagen is indispensable in the HL^24,25^. Consequently, collagen-depletion strategies remained unpopular. At the time, attention was not given to the fact that NaOCl is an oxidizing agent, directly interfering with free-radical addition polymerization, via since the oxygen species it produces can inhibit free radical activity, so inducing an important reduction of bond strengths^26,27^. Furthermore, retention of NaOCl within demineralized dentin affects the resin-dentin interface^25^. This means that the negative results attributed with the removal of demineralized collagen fibrils could have been related to the detrimental effects of the agent used for that effect, rather than to not having collagen in the HL. Such results could have contributed to the unpopularity of the technique.

Interestingly, recent research has focused again on collagen-depletion strategies^28–30^. Indeed, other deproteinizing agents have been explored for the same end, namely enzymatic pre-treatments (i.e., trypsin, bromelain) to breakdown the collagen matrix or non-collagenous proteins in the extracellular matrix (ECM), such as proteoglycans (PGs) glycosaminoglycans (GAGs)^30–32^. These novel approaches have found positive and promising results, which may lead us to the verge of a new bonding approach to dentin. However, a qualitative and quantitative synthesis of preliminary studies whose objective is removing the collagen to alter hybridization, is still lacking. Considering the research that has been published in the matter, the objective of this systematic review was thus to investigate whether a correct collagen-depletion strategy, after acid-etching, could improve the bond strength of resin-based materials to dentin. Considering a systematic review typology, the null hypothesis would be that, based on the body of evidence available, there are no differences in the bond strength of adhesive materials to dentin, when collagen-depletion was carried out after acid-etching, compared to a conventional hybrid layer.

## Materials and methods

### Systematic search

The present systematic review (SR) was carried out following the latest PRISMA (Preferred Reporting Items for Systematic Reviews and Meta-Analysis) statement guidelines and flowcharts. The protocol associated to this systematic review was registered using the international prospective register of systematic reviews (PROSPERO CRD42022303858). The databases used for the electronic search were PubMed/Medline, Scopus, EMBASE and SciELO and the search was performed by two reviewers, working independently. The IADR Abstract Archive was additionally searched for potentially relevant studies using a simple keyword search strategy. For systematic keyword search using the databases above, both MeSH terms and free keywords were used. The combinations used to search the different databases are shown in Table 1.

**Table 1:** Search strategy used and adapted for the four different electronic databases.

Database	Search strategy
PubMed/Medline	Dentin* AND (“collagen removal” OR “collagen depletion” OR “collagen-depletion” OR deprotein* OR bromelain OR trypsin OR papain OR pepsin) AND (self-adhesive cement OR cement OR hydrophobic resin OR adhesive OR DBA OR “dentin bonding agent” OR dental resin)
Scopus	TITLE-ABS-KEY (dentin*) AND ((“collagen removal”) OR (“collagen-depletion”) OR (“collagen depletion”) OR (deprotein*) OR (“pepsin”) OR (“papain”) OR (“trypsin”) OR (“bromelain”)) AND ((“adhesive”) OR (“DBA”) OR (“self-adhesive cement”) AND (“cement”) OR (“experimental resin”) OR (“hydrophobic resin”) OR (“dentin bonding agent”) OR (“dental resin”))
EMBASE	Dentin* AND (“collagen removal” OR “collagen depletion” OR “collagen-depletion” OR deprotein* OR bromelain OR trypsin OR papain OR pepsin) AND (self-adhesive cement OR cement OR hydrophobic resin OR adhesive OR DBA OR “dentin bonding agent" OR dental resin)
SciELO	(dentin) AND ((((((((deprotein*) OR (collagen removal) OR (collagen depletion) OR (collagen-depletion) OR (trypsin) OR (bromelain) OR (pepsin) OR (papain))))))))

Following initial database search, and after paper screening, each primary study included was manually searched for additional relevant papers in the reference list. An online literature visualization tool (https://www.connectedpapers.com/) was also used to identify additional relevant studies not retrieved in the database search. The search period of this SR was between December 2021 and January 2022, with the last search being conducted on the 17 January 2022. No language or publication date restrictions were applied to the systematic search.

### Review question, PICO and inclusion/exclusion criteria

This SR aims to answer the following general question: “Do collagen depletion strategies improve the bond strength of resin-dentin interfaces?”, which subdivides into specific questions, such as: (1) Is the use of sodium hypochlorite succeeded by an antioxidant, after acid-etching, effective in improving the bond strength of resin-dentin interfaces?; (2) are the use of enzymatic pre-treatments, after acid-etching, effective in improving the bond strength of resin-dentin interfaces?

A PICO strategy format was taken in consideration to organize the inclusion and exclusion criteria and to define the research question. PICO was defined as follows: P—permanent human posterior teeth; I—adhesive restorations, performed in dentin, which used a deproteinizing pre-treatment strategy, after acid etching, to remove surface collagen; C—traditional adhesive restorations relying on conventional hybrid layer formation; O—immediate bond strength, usually at 24 h (tensile or shear). The criteria used for inclusion and exclusion of studies was pre-determined and approved by all review members and is summarized in Table 2.

**Table 2:** Inclusion and exclusion criteria used for reference screening.

Inclusion	Exclusion
Human permanent posterior teeth	Radicular dentin or carious dentin
Sound dentin	Studies which simulated erosion or in substrates affected by disease (molar-incisor hypomineralization or amelogenesis/dentinogenesis imperfecta)
Direct restorative procedures	Glass ionomer cements (GICs) or bioactive materials
Restorative procedures featuring a deproteinizing pre-treatment after acid etching	Studies which simulated erosion
Aging in water, artificial saliva or thermocycling	Smear-layer deproteinization (before acid-etching)

Studies were considered ineligible if they did not perform deproteinization after acid-etching, since the scope of this review comprises the removal of the collagen network exposed after acid-etching. Pre-etching strategies generally relate to smear layer deproteinization which is not the present aim. Moreover, regarding the studies which used sodium hypochlorite (NaOCl), it was decided to include only studies that used an antioxidant after application of NaOCl, to counter the oxidizing effect of hypochlorite. Antioxidants such as sodium ascorbate or grape seed extract are able to neutralize reactive species formed through the oxidizing effect of NaOCl, when in contact with dental hard tissues^33,34^. Without the use of an antioxidant, the results of the studies cannot be accurately trusted, as poor bond strength results may be attributed to the oxidizing effect, which affects the polymerization of the resin materials placed after, rather than to the strategy itself. Thus, to have reliable pooled results in the present study, references that did not use an antioxidant following NaOCl application were thus excluded.

### Data collection and data items extraction

In the beginning, after the systematic search, and following the PRISMA flowchart recommendations, references were exported to a reference organization software (Mendeley Desktop for Mac v. 1.19.8), in which title and abstracts were subject to comprehensive screening by two review members, working independently (A.D. and M.B.C.). Duplicate removal was also performed and registered before screening. To resolve disagreements, the opinion of a third reviewer (A.M.A.) was sought and consensus was reached. Further screening and eligibility followed the pre-determined inclusion and exclusion criteria stringently. Reasons for study exclusion were also documented. When full texts could not be accessed, researchers were contacted via a digital platform (www.researchgate.net) or e-mail.

After final selection of references and their inclusion for the systematic review, quantitative and qualitative data were extracted from each record to a Microsoft Excel (v. 16.16; Microsoft, Redmond, WA, USA) spreadsheet containing a form previously made by the two assessors (A.D. and M.B.C.) and approved by all the review team members. Qualitative data extracted for the systematic review included authors, publication date, country, intervention, experimental groups, sample size, materials used, shear or microtensile bond strength outcome time-points, type of aging conducted and main conclusions. As for quantitative data, used in the meta-analysis, all microtensile bond strength results were gathered in the form of means, standard deviation at all time-points and also corresponding statistical inference tests for multi-comparisons. Data was collected independently, by both reviewers (A.D. and M.B.C.), and cross-checked by an additional reviewer (S.S.).

### Quality assessment (risk of bias)

To assess the risk of bias (RoB) of the primary studies included in this review, and since no pre-specified tool exists for in vitro studies, let alone for dental studies, an in vitro evaluation scale was adapted as similarly done by previous authors^35,36^, but organized by domains and including relevant sources of bias for dental materials studies. Sources of bias were grouped in 4 different domains: Domain 1—bias in planning and allocation; Domain 2—bias in specimen preparation; Domain 3—bias in outcome assessment and Domain 4—bias in data treatment and reporting. The risk of bias was individually measured by two reviewers (A.D. and M.B.C.) and confirmed by a third member of the team (A.M.A.). Once again, disagreements were resolved by reaching consensus among the three assessors. A table summarizing the RoB results was made and the RobVis web visualization tool (www.riskofbias.info/welcome/robvis-visualization-tool) was used to build output figures. Grading of cumulative evidence was not performed as a meta-analysis was not possible considering the studies that were included.

### Data analysis

Meta-analytical analyses were not carried out due to the reduced number of studies found for each deproteinization strategy retrieved in the systematic search (NaOCl application or enzymatic agents) and their methodological weaknesses. Due to this, qualitative synthesis was solely adopted in this systematic review.

## Results

### Study selection and summary of studies—SR

**Figure 1:**
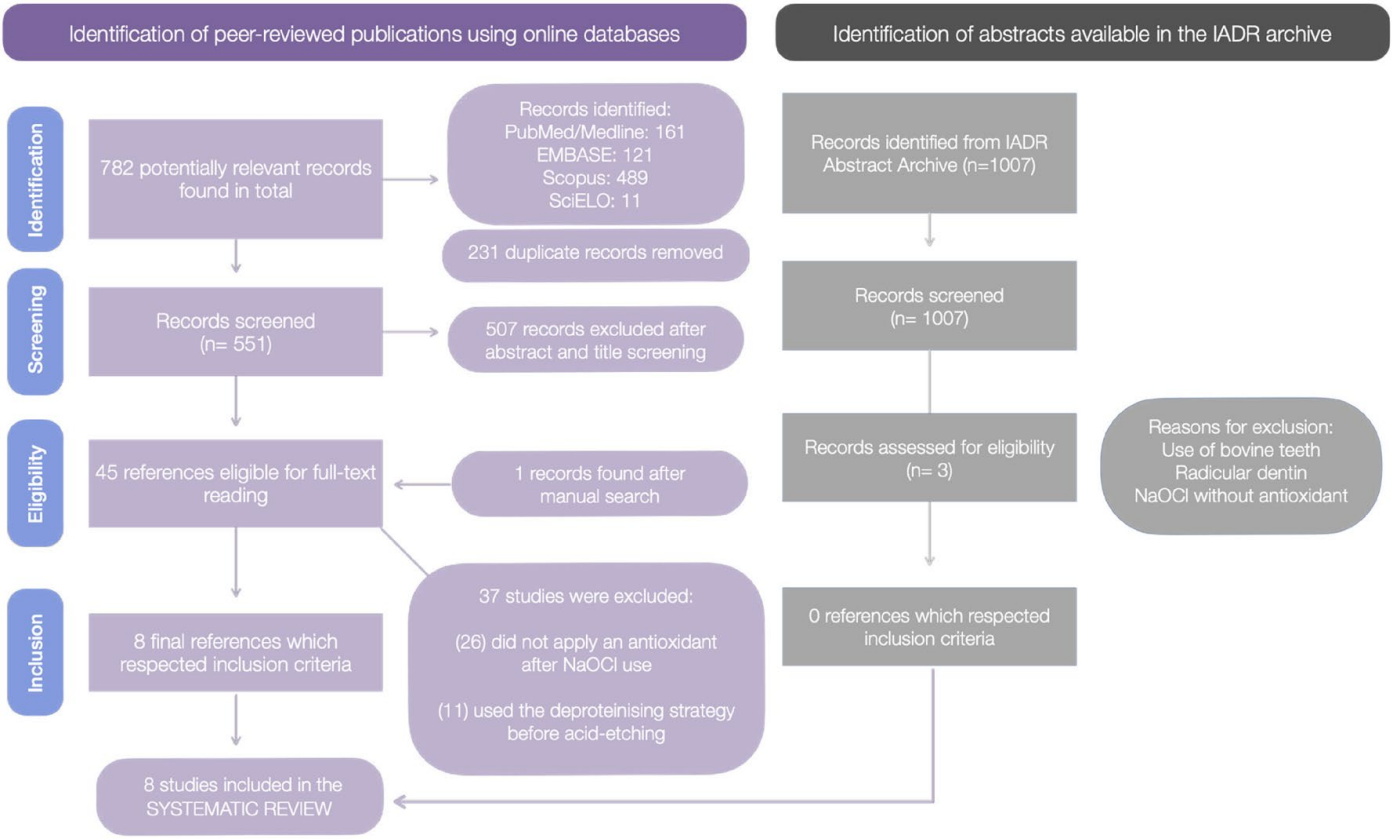
PRISMA workflow chart followed in this SR.

Subsequent to a careful screening of all retrieved references from the systematic search, 46 papers were short-listed for full-text reading. Out of these, 8 studies respected the inclusion criteria and were included in the systematic review^30,37–42^. The PRISMA workflow considered in this SR is depicted in Fig. 1. One additional paper was found through manual search, which was also included in the SR. The remaining excluded papers either applied no antioxidant after using NaOCl or used the deproteinizing strategy before acid-etching (smear layer deproteinization). In one study no bond strength test was performed^32^. The characteristics of these studies are summarized in Table 3.

**Table 3:** Systematic review table summarizing the study characteristics: author/date, country of the study, deproteinizing agent used, sample size, adhesives tested, outcomes measured, whether the strategy improved the bond strength compared to control and final conclusions.

Author	Country	Deproteinizing agent	Sample size per experimental group	Material tested	Outcomes tested	Improved bond strength compared to control?	Conclusion
**Lai et al. (2001) **^37^	**Hong Kong SAR**	**5.25% NaOCl for 1 min + 10% sodium ascorbate for 1 min**	***n* = 4**	**Single bond [SE] (3M ESPE, Seefeld, Germany)** **ExciTE [ER] (Ivoclar Vivadent, Schaan, Liechtenstein)**	**µTBS (24 h)** **TEM, SEM**	**No difference**	**The demineralized collagen matrix was only partially removed by NaOCl. The compromised bond strength of NaOCl seen in other studies may be related to redox issues, rather than to the collagen removal itself**
**Ribeiro et al. (2011) **^82^	**Brazil**	**10% NaOCl for 1 min or 4.25% NaOCl for 45 s + 10% sodium ascorbate for 10 min or 10% ascorbic acid**	***n* = 2**	**All bond 2 [ER] (Bisco, Schaumburg, IL, USA)** **Adper single bond 2 [SE] (3M ESPE, Seefeld, Germany)**	**µTBS (24 h)**	**Material-dependent**	**Deproteinization with NaOCl followed by use of sodium ascorbate or ascorbic acid showed material-dependent trends. Ascorbic acid seemed to be the antioxidant with the best results**
Gao et al. (2014)^38^	China	Chondroitinase ABC for 48 h Trypsin for 48 h	*n* = 7	Adper single bond 2 [SE] (3M ESPE, Seefeld, Germany) Prime and bond NT [ER] (Dentsply Sirona, York, PEN, USA)	µTBS (24 h) SEM	Yes	Proteoglycans participate in the adhesive process in dentin. Their removal increased the immediate µTBS, while removing glycosaminoglycans decreased it
Chauhan et al. (2015)^39^	India	Bromelain for 1 min 5% NaOCl for 1 min	*n* = 10	Adper single bond 2 [SE] (3M ESPE, Seefeld, Germany)	SBS (24 h)	Yes	Bromelain enzyme application was able to improve bond strengths when compared to no treatment or application of 5% NaOCl
Gao et al. (2017)^40^	China	Chondroitinase ABC for 48 h Trypsin for 48 h	*n* = 7	Adper single bond 2 [SE] (3M ESPE, Seefeld, Germany) Prime and bond NT [ER]	µTBS (24 h and 1 year) SEM	Yes	Removal of proteoglycans increased the immediate bond strength and its longevity (after storage in artificial saliva for up to 1 year)
Farina et al. (2020)^30^	USA	1 mg/mL Trypsin for 24 h	*n* = 10	Scotchbond Universal [U] (3M ESPE, Seefeld, Germany) Prime and bond NT Hydrophilic experimental adhesive Hydrophobic experimental adhesive	µTBS (24 h) Contact angle	Yes	Removal of proteoglycans greatly enhanced wettability and immediate bond strengths of hydrophobic mixtures
Khan et al. (2020)^41^	India	Bromelain 5% NaOCl 10% NaOCl	*n* = 40	Prime and bond NT [ER] (Dentsply Sirona, York, PEN, USA)	SBS (24 h)	Yes	The application of bromelain enzyme showed better bond strengths than 5/10% NaOCl and was significantly better than not performing deproteinization
Khatib et al. (2020)^42^	India	8% Bromelain for 1 min 8% Papain for 1 min 5.25% NaOCl for 1 min	*n* = 20	N/A	µTBS (24 h)	Yes	All groups performed better than not carrying out deproteinization. Bromelain enzyme application achieved the highest bond strengths

Studies marked in bold are NaOCl strategies, while the ones marked in white are the enzymatic deproteinization studies.

Six studies dealt with enzymatic treatments, such as the application of trypsin and/or chondroitinase ABC, to remove PGs and GAGs, or bromelain as a collagenase^30,38–42^. Two studies involved the application of varying concentrations of NaOCl (4.25, 5.25 or 10%) followed by the application of an antioxidant. It is important to highlight that not a single study reported a decrease in bond strength, when a deproteinizing technique was used. Deproteinizing strategy application times varied from 1 min (NaOCl) up to 48 h (trypsin).

### RoB analysis of the studies

**Figure 2:**
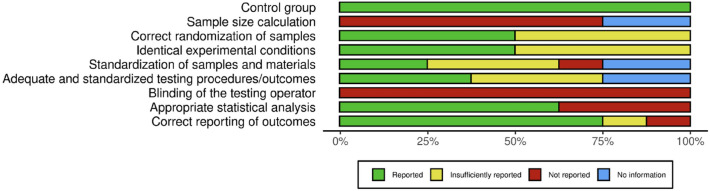
RoB analysis plot summary showing the distribution of RoB classifications across different sources of bias.

A RoB analysis of the 8 studies shown above is presented in Table 4. An illustrative image of the RoB overall outcome, considering all sources of bias for all studies, can be seen in Fig. 2. Overall, studies included in this SR were classified as having a moderate to high risk of bias. Sources of bias such as sample size calculations (D1) and blinding of the testing operator (D3) were not reported in any study. Other important sources of bias, such as identical experimental conditions and standardization of samples and materials (D2) were only sufficiently reported in 50% and 25% of the studies, respectively.

**Table 4:** RoB analysis for the 8 studies included in this SR, shown in Table 3.

Author	D1: bias in planning and allocation			D2: bias in specimen preparation		D3: bias in outcome assessment		D4: bias in data treatment and reporting	
	Control group	Sample size calculation	Correct randomization of samples	Identical experimental conditions	Standardization of samples and materials	Adequate and standardized testing procedures/outcomes	Blinding of the testing operator	Appropriate statistical analysis	Correct reporting of outcomes
**Lai et al. (2001)**	**Reported**	**Not reported**	**Not reported**	**Reported**	**Reported**	**Reported**	**Not reported**	**Reported**	**Reported**
**Ribeiro et al. (2011)**	**Reported**	**Not reported**	**Reported**	**Reported**	**Not reported**	**Insufficiently reported**	**Not reported**	**Not reported**	**Reported**
Gao et al. (2014)	Reported	N/A	N/A	Reported	N/A	N/A	N/A	Reported	Reported
Chauhan et al. (2015)	Reported	Not reported	Not reported	Insufficiently reported	Insufficiently reported	Insufficiently reported	Not reported	Reported	Reported
Gao et al. (2017)	Reported	N/A	N/A	Reported	N/A	N/A	N/A	Reported	Reported
Farina et al. (2020)	Reported	Not reported	Not reported	Insufficiently reported	Reported	Reported	Not reported	Reported	Not reported
Khan et al. (2020)	Reported	Not reported	Not reported	Insufficiently reported	Insufficiently reported	Insufficiently reported	Not reported	Not Reported	Insufficiently reported
Khatib et al. (2020)	Reported	Not reported	Not reported	Insufficiently reported	Insufficiently reported	Reported	Not reported	Not reported	Reported

NaOCl studies are shown in bold.

### Excluded studies using NaOCl without an antioxidant

To map the retrieved studies (26) where NaOCl was used as a deproteinizing agent after acid etching, but without a subsequent application of an antioxidant, a table summary with their highlights was constructed and shown (Table 5). Such a table can confirm the conflicting results reported in the literature during the last 20 years, since it is possible to observe that an improvement in bond strengths following the use of NaOCl varied upon concentration, application time, the adhesive type, and its chemistry.

**Table 5:** Data from the studies excluded (26) in the SR which used NaOCl as a deproteinizing strategy after acid etching, without an antioxidant, and that tested tensile or shear bond strength as an outcome.

Author and date	Deproteinizing agent/concentration	Materials	Improved bond strength compared to control?	Conclusion
Saboia et al. (2000)^63^	10% NaOCl for 1 min	Prime and bond NT [ER] (Dentsply Sirona, York, PEN, USA)	Similar	The results may suggest that collagen removal improves the bond strength for this acetone-based adhesive system but other systems should be investigated
Toledano et al. (2002)^83^	5% NaOCl for 2 min	Prime and bond 2.1 [ER] (Dentsply Sirona, York, PEN, USA)	Similar	Deep and superficial dentin that was deproteinized using 5% NaOCl resulted in similar SBS to non-deproteinized control
Osorio et al. (2002)^84^	5% NaOCl for 2 min	Adper single bond [ER] (3M ESPE, Seefeld, Germany)	No	Adverse chemical interactions could have occurred between the remnant collagen matrix and/or mineralized dentin after NaOCl treatment. There is no additional advantage in using NaOCl treatment with this adhesive
Munksgaard (2002)^85^	0.5% NaOCl for 1 h	Clearfil liner bond [SE] (Kuraray Noritake, Tokyo, Japan) ExciTE [ER] (Ivoclar Vivadent, Schaan, Liechtenstein) Optibond FL [ER] (Kerr, Orange, USA) Optibond Solo Plus [SE] (Kerr, Orange, USA) Prime and bond NT [ER] Scotchbond 1 XT [SE] (3M ESPE, Seefeld, Germany)	Yes	In most of the studies, higher or similar bond strengths were observed when adhesives were tested on deproteinized dentin compared with normal etched dentin
Uceda-Gómez et al. (2003)^86^	10% NaOCl for 1 min	N/A	No	The application of sodium hypochlorite following dentin acid etching may reduce bond strengths
De Souza et al. (2005)^87^	5% NaOCl for 2 min	Adper single bond [ER] Prime and bond NT [ER] One coat bond [SE] (Cóltene/Whaledent, Alstatten, Switzerland)	No	The bonding performance on deproteinized dentin surfaces depended on the characteristics of each adhesive system, as well as the on the adhesive dentin specificity related to the oxidant effect of NaOCl
Silva et al. (2007)^88^	10% NaOCl for 1 min	Prime and bond NT [ER] Clearfil SE bond [SE] (Kuraray Noritake, Tokyo, Japan) Scotchbond MP plus [SE] (3 M ESPE, Seefeld, Germany)	Material-dependent	The influence of dentin deproteinization was dependent on the dentin bonding system formulation
Uceda-Gómez et al. (2007)^89^	10% NaOCl for 1 min	Single bond [SE] One-step [SE]	No	The use of 10% NaOCl, after acid etching, did not improve the immediate and the long-term resin-dentin bond strength
Erhardt et al. (2008)^90^	10% NaOCl for 2 min	Clearfil SE bond [SE] (Kuraray, Tokyo, Japan) One-up bond F [SE] (Tokuyama, Tokyo, Japan) Etch and Prime 3.0 [SE]	Yes	The one-step self-etch adhesives benefited from the deproteinization technique undertaken with NaOCl
Saboia et al. (2008)^91^	10% NaOCl for 1 min	XP-Bond [ER] (Dentsply Sirona, York, PEN, USA)	No	Authors reported that the role of collagen fibrils seems fundamental for bonding with XP-Bond to dentin, as decreased immediate bond strength and reduced bond stability over time was found on collagen-depleted dentin
Silva et al. (2009)^92^	10% NaOCl	DenTASTIC Uno [SE] (Pulpdent, Watertown, MA, USA)	Yes	The deproteinization protocol resulted in an improvement of bond strengths in 5 out of 6 of the adhesives tested in the study
Braz et al. (2009)^93^	5% NaOCl for 2 min	Adper promp L-pop [SE] (3M ESPE, Seefeld, Germany) Adhese [SE] (Ivoclar Vivadent, Schaan, Liechtenstein)	Yes	Deproteinization contributed favorably to the bond strength of the adhesive systems to dentin
Baseggio et al. (2009)^94^	10% NaOCl for 1 min	Adper single bond [ER] (3M ESPE, Seefeld, Germany)	No	Dentin deproteinization with NaOCl and oxalate significantly compromised both the adhesive bond strength and microleakage
Sacramento et al. (2011)^64^	0.5% NaOCl for 30 min	Adper single bond 2 [ER] Clearfil protect bond [SE] Adper prompt L-pop [SE]	Yes	The etch-and-rinse and the two-bottle self-etching AS produced the highest microtensile values irrespective of prior NaOCl irrigation
Chaharom et al. (2011)^95^	5.25% NaOCl for 5 min	Clearfil S3 Bond [SE] (Kuraray Noritake, Tokyo, Japan)	No	The use of NaOCl reduced the shear bond strength of fifth- and seventh-generation adhesive resins to dentin and there was no difference in the shearing bond strength of both adhesive resins
De Souza et al. (2011)^96^	5% NaOCl for 2 min	Adper single bond 2 [ER]	Yes	The collagen-depletion technique provided an improved bond performance for the self-adhesive cement Rely X Unicem, and had no negative effect on the other cement systems studied
Aguilera et al. (2012)^97^	5% NaOCl for 2 min	Prime and bond NT [ER]	No	The application for 2 min of 5% NaOCl did not improve bond strength
Lisboa et al. (2013)^98^	5% NaOCl for 2 min	RelyX Unicem (3M ESPE, Seefeld, Germany) BisCem (Bisco, Schaumburg, USA) cement	Yes	Deproteinization improved the bond strength of BisCem to dentin but did not improve the performance of RelyX Unicem when compared to untreat- ed dentin specimens
Faria-e-Silva et al. (2013)^99^	10% NaOCl for 1 min	Experimental adhesives containing a Bis-GMA/HEMA blend diluted in ethanol (7.5, 15 or 30 mass%) or acetone (15, 30 or 60 mass%) (low, medium or high solvent content, respectively)	Yes	The deproteinization protocol improved the bond strength of the experimental materials
Francescantonio et al. (2015)^100^	10% NaOCl for 1 min	One-step plus (Bisco Inc.) Clearfil photo bond (Kuraray Noritake, Tokyo, Japan) Clearfil SE bond	No	10% NaOCl changed the morphology of the bonding interfaces and its use with etch-and-rinse adhesives reduced the dentin bond strength
Montagner et al. (2015)^101^	10% NaOCl for 1 min	Adper single bond 2 [ER] Clearfil SE bond [SE] Adper SE Plus [SE] G-Bond (GC Corp., Tokyo, Japan)	Similar	The deproteinization pretreatment showed similar bonding effectiveness to the conventional adhesive technique. The dentin region plays a rule on the bond strength values
De Souza et al. (2016)^102^	5% NaOCl for 2 min	RelyX ARC (3M ESPE, Seefeld, Germany) Adper single bond 2 [ER]	Yes	The dentin deproteinization improved the initial bond strength of both cements tested, but, after thermocycling, this technique seemed only effective for RelyX U200
Pucci et al. (2016)^103^	10% NaOCl for 1 min	DenTASTIC Uno [SE] Prime and bond NT [ER] Single bond [SE]	Yes	The use of 10% NaOCl deproteinization protocol may improve the bond durability in adhesive restorations
Dikman and Tarim (2018)^104^	5.25% NaOCl for 30 s	Adper single bond [ER] Clearfil SE bond [SE] Xeno III [SE] (Dentsply Sirona, York, PEN, USA)	No	Different irrigants had distinct effects on bonding of different adhesives. Sodium ascorbate after NaOCl could restore compromised bond strengths
Rodrigues et al. (2018)^62^	5% NaOCl for 2 min	RelyX U200 (3M ESPE, Seefeld, Germany) MaxCem Elite (Kerr, Orange, USA)	Yes	Dentin deproteinization prior acid etching increased the µTBS of both cements at 24 h, but no differences in RU groups were found after load cycling
Nima et al. (2020)^65^	10% NaOCl for 1 min	Gluma 2Bond [ER] (Heraeus Kulzer, Hanau, Germany) One-step [SE] (GC Corporation, Tokyo, Japan)	No	Bond strength of deproteinized dentin was dependent on the adhesive system composition and NaOCl accelerated aging promoted decreased bond strength and further degradation than water storage for 1 year

Improvement of dentin bond strength was categorized as “similar”, if there were no differences, “no” if reduced bond strengths were reported and “yes” if improvement was achieved.

## Discussion

Numerous attempts have been made to develop resistant hybrid layers, able to withstand degradation processes in the oral cavity by limiting hydrolysis mechanisms and inhibiting enzymes which breakdown the unsupported organic content. Most of them have only been able to retard the problem rather than completely resolve it^43,44^. Thus, innovative, radical treatments and approaches are still needed^7^. Considering this, the objective of this systematic review was to investigate the evidence on the benefit of removing the acid-etched exposed collagen, by using deproteinizing agents, on the bond strength of adhesive materials to dentin.

Dentin is a highly mineralized substrate with a particular difference—it contains, in addition to a mineral apatite-based phase (mainly hydroxyapatite), a high volume of organic content that range from 30 to 50% of its total vol%; it is mainly composed of type-I collagen, and as much as 20 vol% of water^45,46^. Water molecules are substantially retained by water-binding proteins such as PGs^30,31^. To etch dental substrates, phosphoric acid (PA) has been preferentially used for more than 40 years^47^. In dentin, the recommended etching time using PA has been limited to 15 s^46^. This exposes a 5–8 μm dense collagen network devoid of hydroxyapatite minerals, which serves as a target for the priming agent present in modern adhesive systems^48^. However, deproteinization carried out after acid etching would remove this exposed collagen network, leaving a slightly roughened hydroxyapatite surface that was hidden underneath, with shallow pits thought to be receptable to resin-based materials^20^. This layer would therefore resemble the bonding mechanism achieved in enamel, which is virtually free from organic content due to its nature. Some authors have named this a “reverse hybrid layer”, since the collagen is no longer infiltrated by the resin-based materials, but rather the resin monomers instead occupy the space where the collagen was originally^20,49^. NaOCl is also able to solubilize underlying fibers, further contributing to the creation of submicron porosities that foster the retention of polymerized resin monomers^49^.

The term deproteinization encompasses not only collagen-depletion strategies but also smear layer deproteinization techniques, which should be distinguished from the former. With smear-layer deproteinization, agents such as NaOCl or hypochlorous acid (HOCl) are used as a surface pre-treatment to remove the organic portion of the smear layer, that generally contains not only organic residues but also residual hydroxyapatite, rotatory instrument debris and bacteria^25^. This was not the aim of the present review, as such technique is used to improve and strengthen subsequent hybridization of self-etch adhesives to dentin^50,51^.

Since a meta-analysis was not possible given the scarce number of studies found and their associated risk of bias, it cannot be possible to answer the review question with quantifiable confidence, although some important conclusions can be drawn, however. Low quality evidence suggests that there may be a beneficial effect of using deproteinizing agents to increase the immediate bond strength to dentin. None of the studies found a detrimental effect of deproteinizing agents in bonding outcome, when compared to a traditional hybrid layer mechanism, considered to be the control comparison in all studies that were included, as can be seen from the evidence gathered in Table 3. This had already been reported in a clinical study with a 5-year follow-up, although the authors did not counter the oxidizing effect of NaOCl^52^. Yet, the impact of these results cannot be ignored. Adhesive restorations performed in cavity preparations that involve dentin surfaces have relied on the formation of a hybrid layer, since it was first seen by John McLean in 1952^53^, and later named and described by Nakabayashi in the early 1980’s^3,54^. Proving that collagen may not be as indispensable as once thought is daunting. A shift in this philosophy is cumbersome but may be required.

Excluding collagen from the bonding layer can be from some points of view advantageous for three concrete reasons: (1) it greatly reduces the technique sensitivity in bonding to dentin, related to its moisture state and the primer used (is it too dry? is it too wet? have I evaporated the solvent enough?)^55,56^; (2) it increases the longevity of the restorative procedure, since there is less water, less incomplete spaces that were not filled by the adhesive and there will be no enzyme-mediated collagen degradation^7,57^; finally (3) it provides a favorable substrate, increasing the wettability for hydrophobic resin mixtures to bond to^27,30^, which were otherwise too viscous to infiltrate the collagen network. In turn, this hydrophobicity also reduces chances of hydrolytic degradation happening at the resin-dentin restorative interface over time.

NaOCl is chemical compound widely used as an endodontic irrigant, known for its non-specific proteolytic and antibacterial activity against the organic layer present in radicular dentin^58^. Deproteinization accomplished with NaOCl at clinically relevant times, in percentages between 2.5 and 10%, results in an only partial removal of the collagen fibrils^59,60^. If a collagen-depletion technique is to be considered, leaving this residual organic content bound to the bondable layer may not be entirely desirable. In fact, such partial collagen depletion may also be responsible for some of the material-dependent results seen over the years. What can be clearly seen from Table 5, are the vast differences in the percentages and application times of NaOCl used. These aspects most likely influence not only the extent of the proteolytic activity—how much collagen is dissolved, but also the impact of the redox effect^61^, detrimental to resin-based materials dependent upon the generation of free-radicals for polymerization mechanisms. Furthermore, the rinsing protocol after the use of NaOCl will most likely also impact the detrimental effect of this agent since ineffective rinsing protocols will not get rid of the residual NaOCl. From the studies in Table 5, many disparities were also found regarding the rinsing protocols^62–65^. Prolonged use of NaOCl may also lead to alterations of the elastic modulus of dentin, making it more brittle and prone to cohesive fracture^66^.

Besides from NaOCl, other alternative irrigants able to dissolve organic content have been proposed and studied in the endodontic field over the years^67–69^. Irrigants such as calcium hypochlorite or chlorine dioxide have been for their organic dissolution capacity^70,71^. In fact, 5% chlorine dioxide has been reported to show interesting and comparable results, to NaOCl, in bond strength outcomes^69^. But limited evidence concerning these alternative oxidizing solutions is available.

Regarding the enzymatic strategies, interestingly, a recent SR and meta-analysis evaluated the role of deproteinizing agents on the bond strength to enamel, used before and after acid etching. Indeed, Fernández-Barrera et al. found that deproteinizing after acid etching enamel did not increase bond strengths, while the opposite did^72^. Papain-based agents were effective in improving the bond strength of resin-enamel interfaces. In the present SR, several different enzymatic agents were used in the eligible studies: bromelain, chondroitinase ABC, papain, or trypsin, with each having their own different targets. Bromelain is a plant protease obtained from the pineapple stem, broadly used in the food industry and able to catalyze the hydrolysis of proteins into amino acids^73^. Its capacity to promote collagen hydrolysis has been verified experimentally^74,75^. Similarly, papain is obtained from papaya and is a cysteine protease known for its specificity, commonly used in atraumatic dental procedures as a chemo-mechanical method to remove caries-affected substrate^73^. Bromelain was applied for 1 min in the study of Chauhan et al. and Khatib et al., with experimental groups achieving better results than the non-deproteinized control^39,42^. Another study which undertook SEM analysis of the surface-treated dentin, found that bromelain treatment for 1 min resulted in a surface free of collagen remnants when compared to 10% NaOCl^32^. The concentration of bromelain was not reported in three studies^32,39^. Papain is already used in caries-affected substrates, although its collagen degradation ability in acid-etched dentin, during a short application period, might be questionable. Still, future studies are needed to consolidate these findings and to determine optimal concentrations and application times of enzymatic solutions.

Removal of PGs and GAGs by virtue of trypsin or chondroitinase ABC digestions was also assessed in three studies included in this SR^30,38,40^. PGs are proteins that are heavily glycosylated, formed by core protein that have one or more GAG chains. They are water-binding proteins that regulate the hydraulic mechanical support system to the collagen network of the ECM in dentin^76^. Removing this excess intrinsic water, by cleaving these proteins, can thus aid wettability and interaction of materials with the surface. To cleave the proteins, chondroitinase ABC or trypsin have been used. While chondroitinase cleaves GAGs, trypsin, in contrast, can digest the protein core releasing the GAG chains. Trypsin may also remove other non-collagenous components of the ECM, that are present in dentin^30^.

It has been shown that trypsin digestion leads to an increased susceptibility of organic tissue degradation, promoting further proteolysis^76^. Gao et al. pioneered the study that first removed GAGs and PGs in dentin, to assess resulting bond strengths. These researchers found that upon removal of PGs, bond strengths were improved, while the opposite happened when GAGs were removed^77^. Farina et al., further to this, showed that a 24 h protocol of trypsin digestion, contrarily to the 48 h attempted by Bedran-Russo et al.^31^ in bovine teeth (excluded from this SR), led to a significant increase in the bond strength of a hydrophobic adhesive blend. This is a result of significant importance^30^. Viscous and hydrophobic resin mixtures used in adhesive dentistry (i.e., flowable or self-adhesive composites), bonded on their own, without a dental adhesive, have always struggled in securing acceptable bonding to dentin up to this day^78–80^.

One of the major gaps found in the evidence was that long-term bond strength data is scarce and largely unavailable. Studies featuring aged experimental groups are not only necessary, but fundamental in the future, to confirm that a collagen-depletion strategy may in fact stabilize bond degradation, eliminating the unpredictability associated to collagen. In fact, an optimal deproteinization strategy is still missing and more studies are required to reach a consensus. Specifically, it is pertinent for further studies to determine which is the best agent, its concentration, and a clinically relevant time frame at which it can be applied to dentin, to secure successful immediate bonding properties and their longevity. All of which are possible in coming studies.

To conclude, the present findings suggest that an effective collagen-depletion protocol will most likely increase immediate bond strengths. Even more importantly, a correct protocol can improve wettability to dentin surfaces depleted of collagen^81^, while enabling a greater chance in increasing bonds of hydrophobic blends to dentin.

## Conclusion

Although a meta-analysis was not possible due to a reduced number of studies found and concerns regarding their methodological quality, collagen-depletion strategies in dentin, especially when enzymes are used, may improve immediate bond strengths. Only two studies that used NaOCl followed by an antioxidant agent were retrieved. Additional research is required to find the best agent, its concentration and application time. Further studies are needed to consolidate these findings and to determine the role of collagen-depletion strategies on the long-term bond strength. Such strategies would be particularly important to enable bonding of hydrophobic materials and mixtures.
